# Supplementary material for: EyeHex toolbox for complete segmentation of ommatidia in fruit fly eyes
Source: Biol Open. 2025 Jun 23;14(6):bio061962. doi: 10.1242/bio.061962 (PMC12233063; doi:10.1242/bio.061962)
Supplement: Supplementary information [file biolopen-14-061962-s1.pdf]

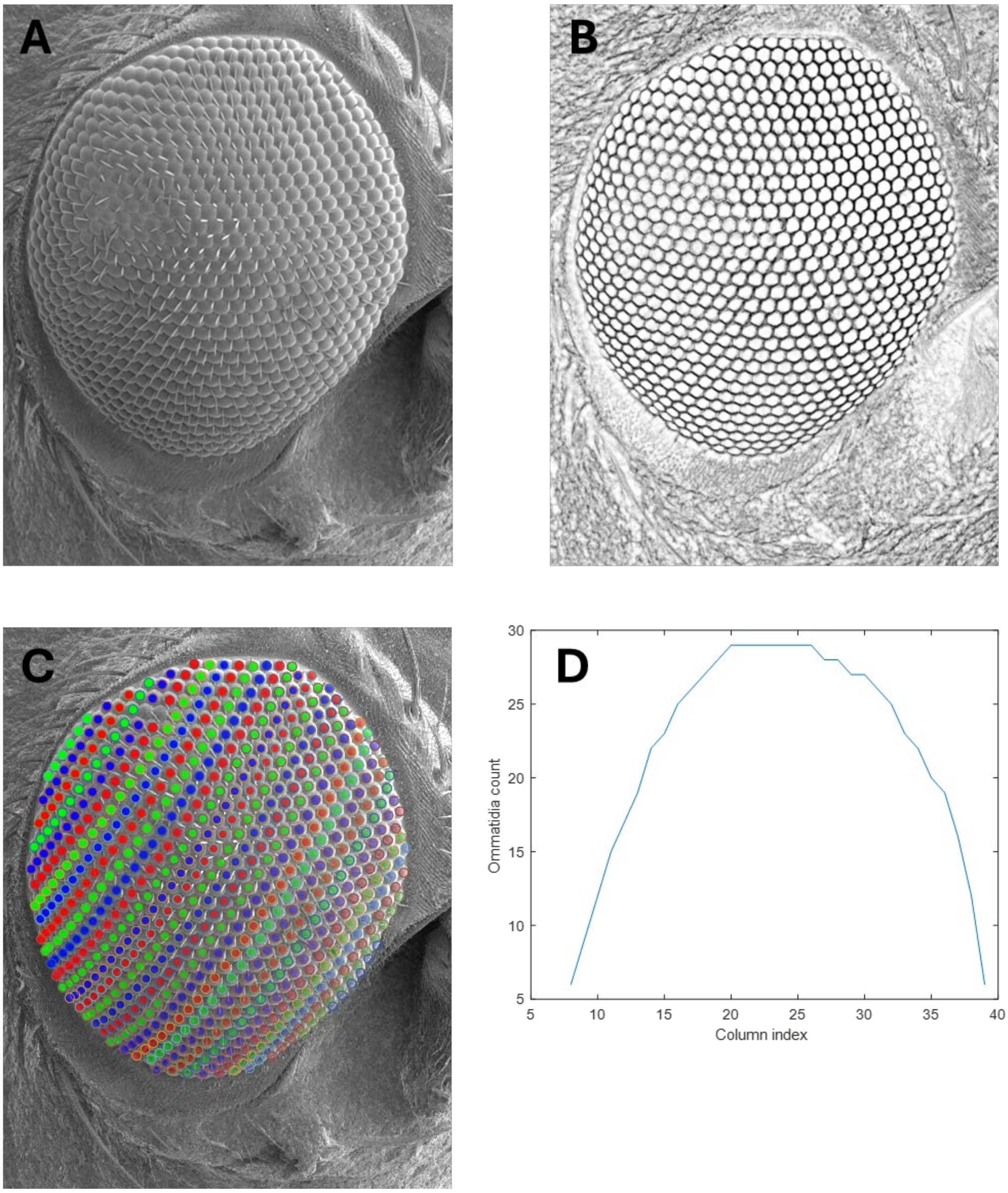

Fig. S1. EyeHex analysis of a SEM image of a Hikone-AS eye *(*data from *Ramaekers et al., 2019).*

For each example SEM image, we show: **(A)** the original image; **(B)** the probability map of ommatidia location generated by the machine-learning module; **(C)** the anterior to posterior columns of detected ommatidia, separated by color and progressive dimming; **(D)** ommatidia count in each column from posterior to anterior.

Biology Open • Supplementary information

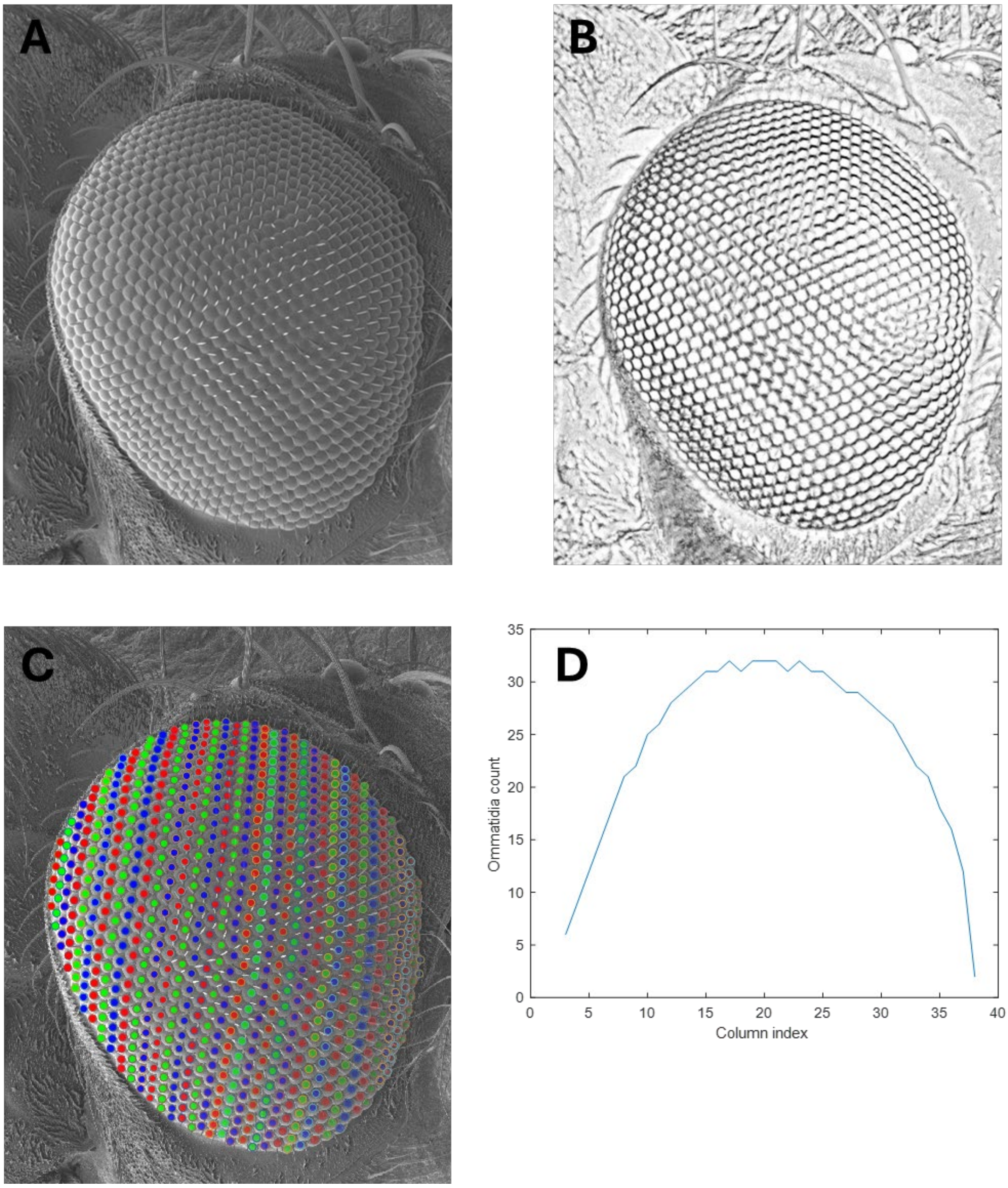

Fig. S2. EyeHex analysis of a SEM image of a Canton-S eye (data from *Ramaekers et al., 2019*).

For each example SEM image, we show: **(A)** the original image; **(B)** the probability map of ommatidia location generated by the machine-learning module; **(C)** the anterior to posterior columns of detected ommatidia, separated by color and progressive dimming; **(D)** ommatidia count in each column from posterior to anterior.

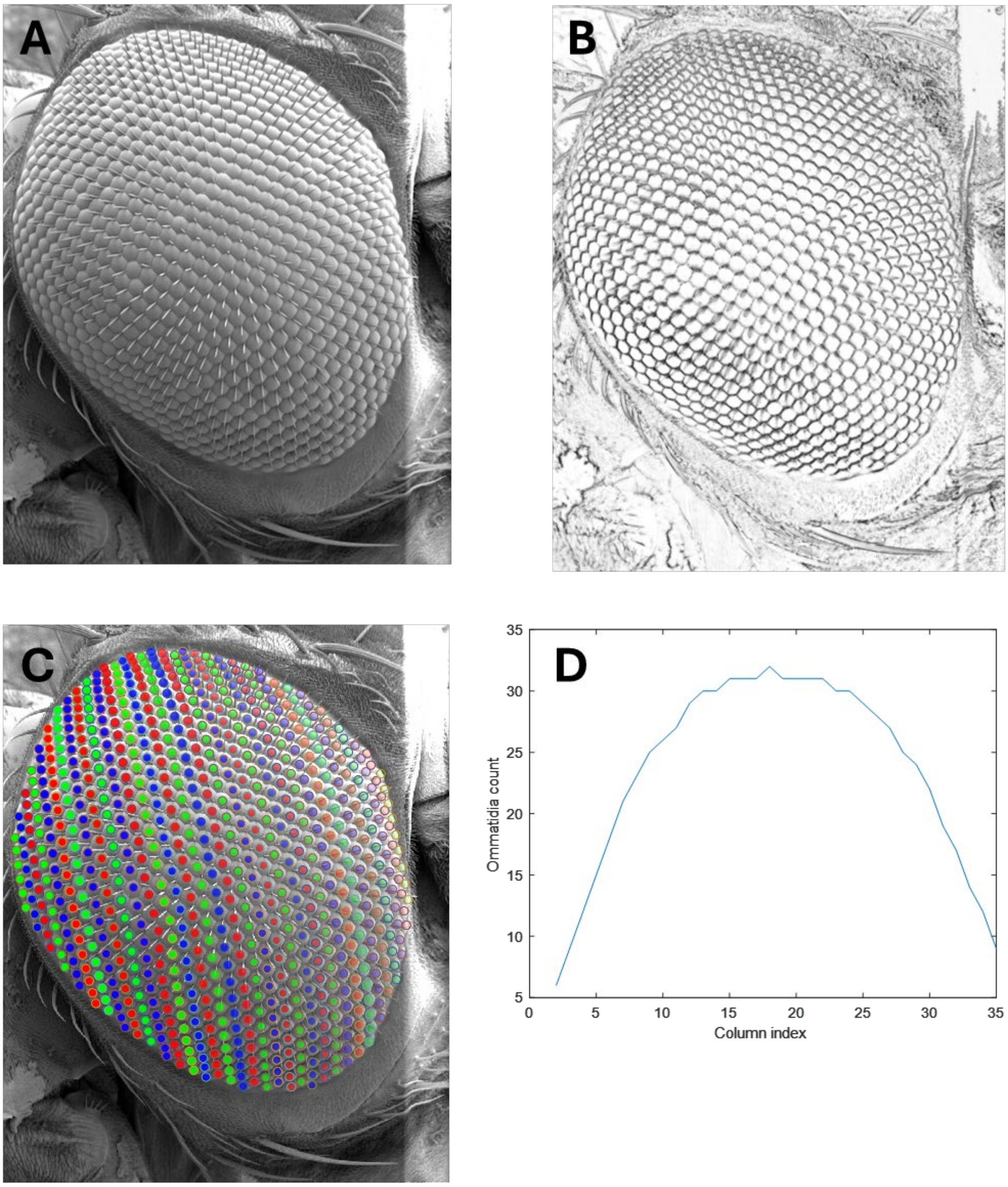

Fig. S3. **EyeHex analysis of a SEM image of a DGRP-208 eye** *(*data from *Ramaekers et al., 2019).*

For each example SEM image, we show: **(A)** the original image; **(B)** the probability map of ommatidia location generated by the machine-learning module; **(C)** the anterior to posterior columns of detected ommatidia, separated by color and progressive dimming; **(D)** ommatidia count in each column from posterior to anterior.

Biology Open • Supplementary information

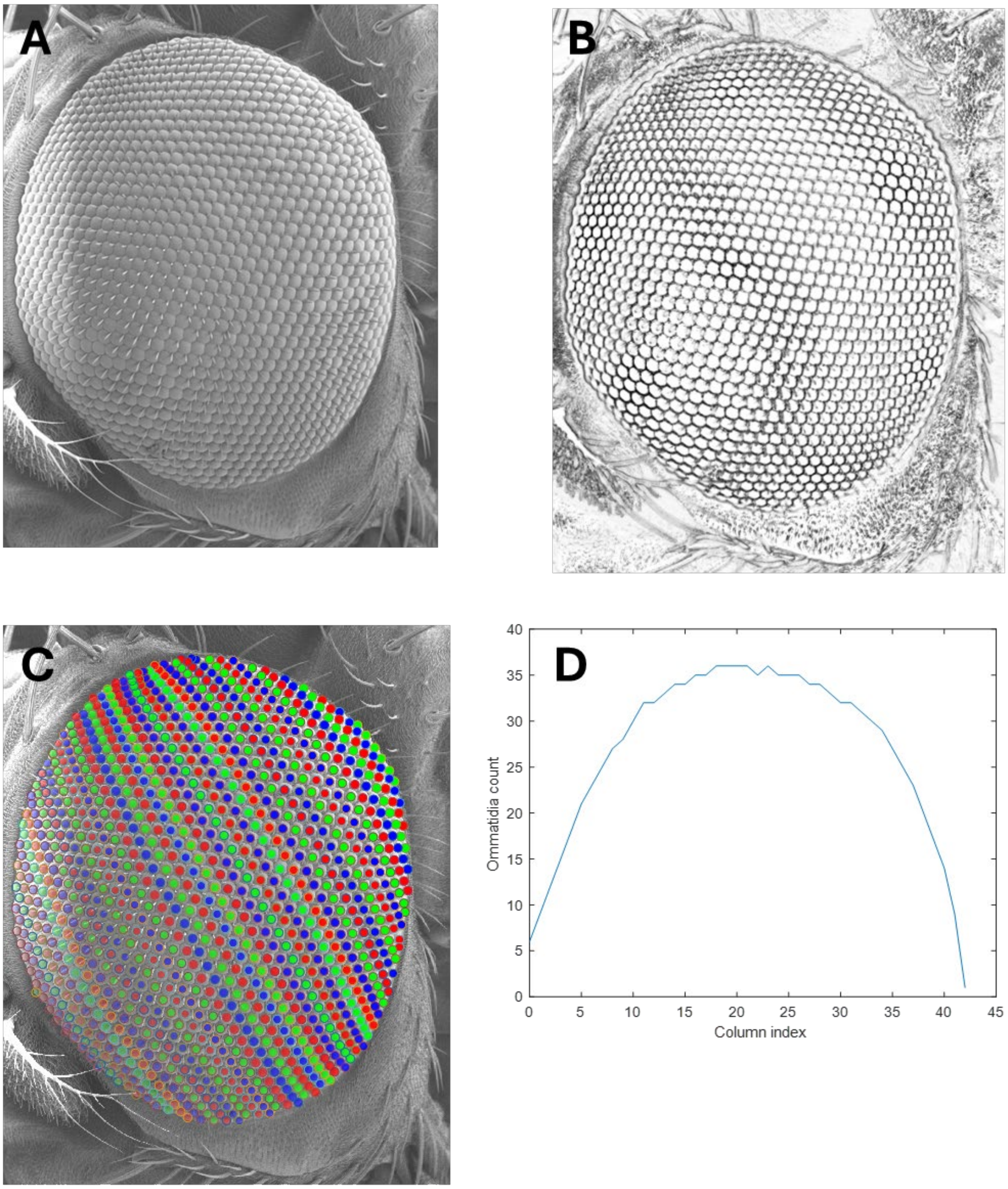

Fig. S4. EyeHex analysis of a SEM image of a *Drosophila pseudoobscura* eye *(*data from *Ramaekers et al., 2019).*

For each example SEM image, we show: **(A)** the original image; **(B)** the probability map of ommatidia location generated by the machine-learning module; **(C)** the anterior to posterior columns of detected ommatidia, separated by color and progressive dimming; **(D)** ommatidia count in each column from posterior to anterior.

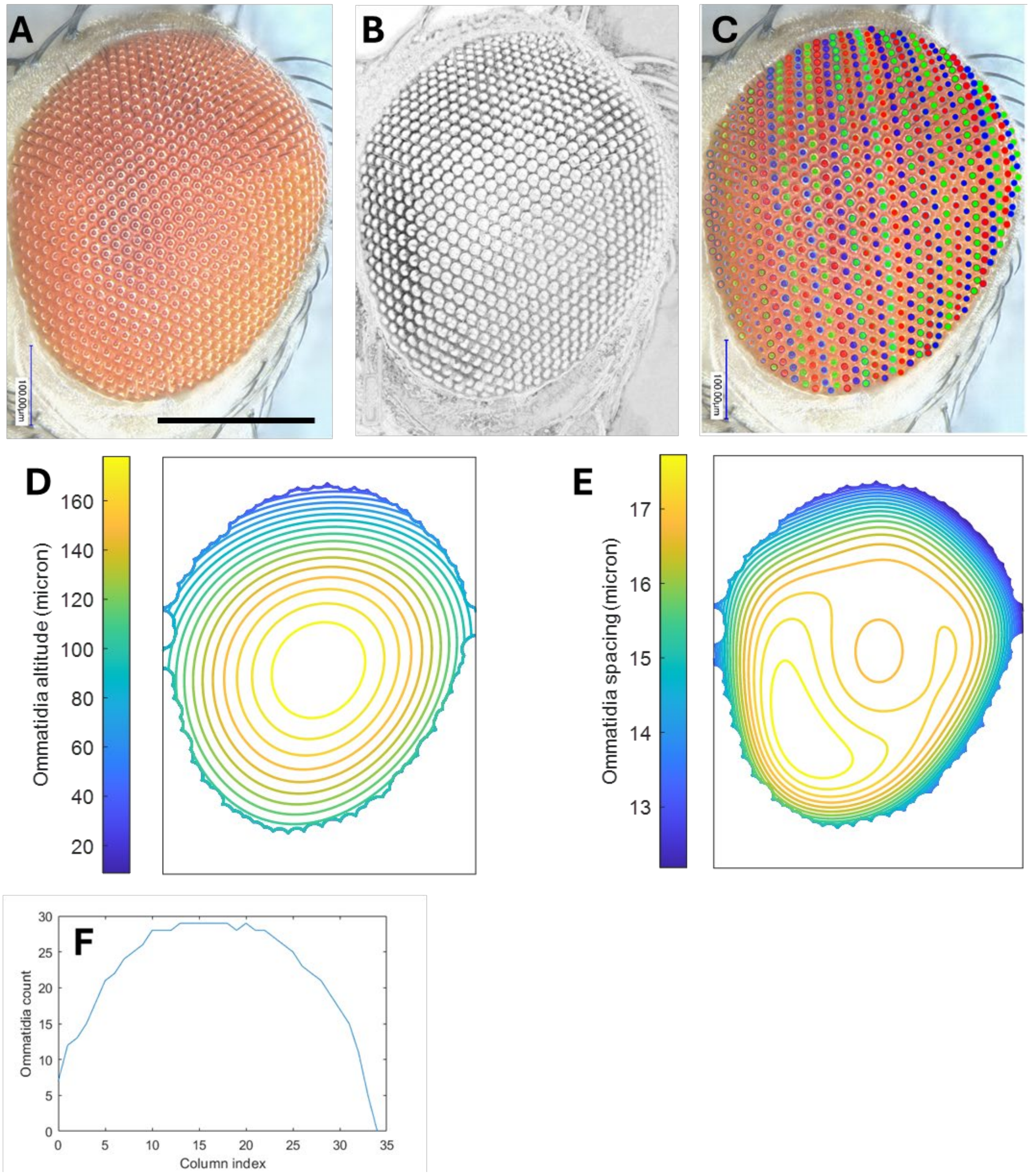

Fig. S5. EyeHex morphological analysis of a brightfield image of an Hikone-AS eye (data**:** this study).

For each example multi-focus image, we show: (**A**) the focused 2D compound image, scale bar: 200 microns; (**B**) the probability map of ommatidia location generated by the machine-learning module; (**C**) the anterior to posterior columns of detected ommatidia, separated by color and progressive dimming; (**D**) a contour map of ommatidia altitude (in micron, calculated relative to the lowest focal plane); (**E**) a contour map of estimated spacing between individual ommatidia (in micron), (**F**) ommatidia count in each column from posterior to anterior.

Biology Open • Supplementary information

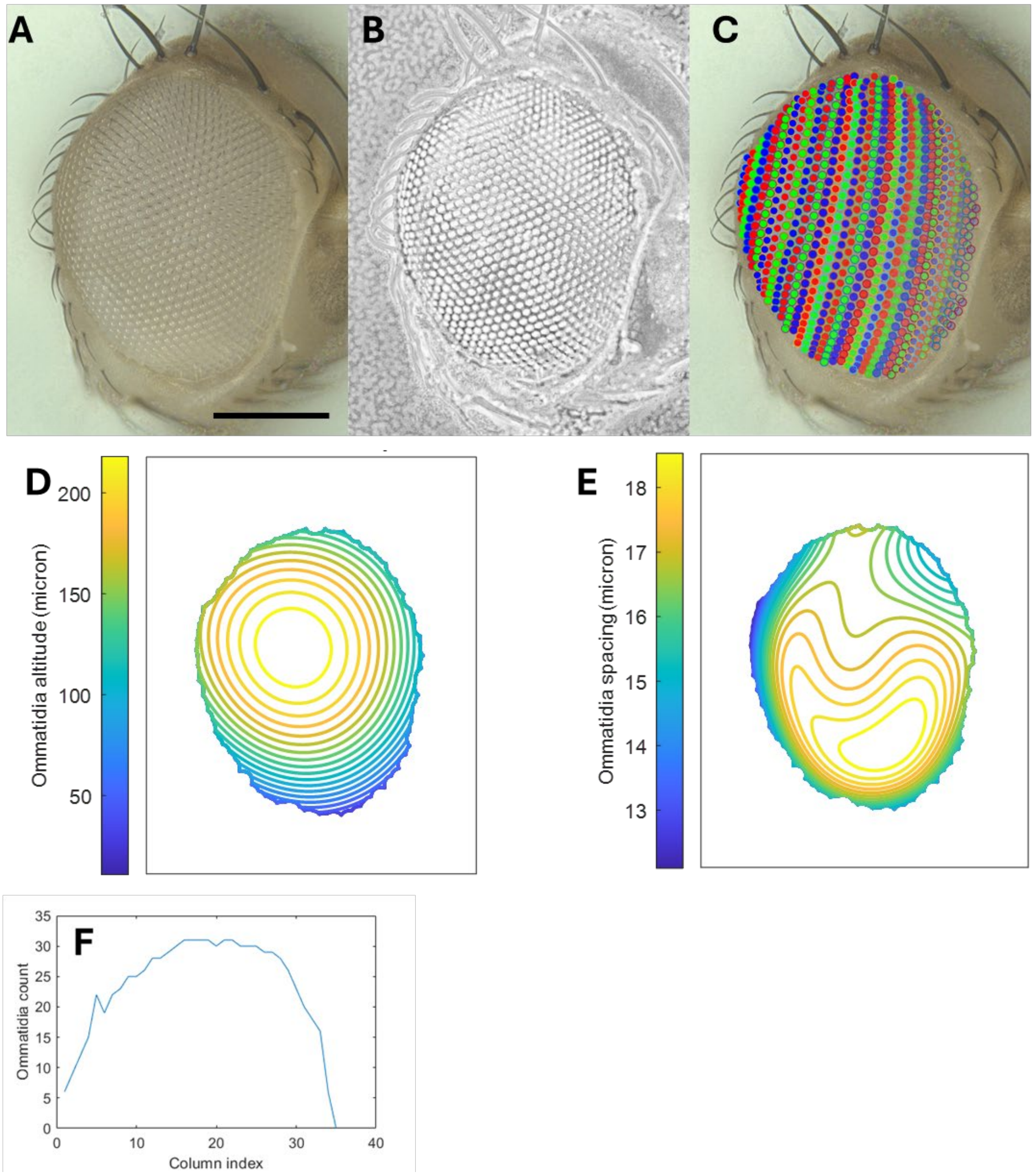

Fig. S6. EyeHex morphological analysis of a brightfield image of a WTJ2 eye (data from *Ramaekers et al., 2019*).

For each example multi-focus image, we show: (**A**) the focused 2D compound image, scale bar: 200 microns; (**B**) the probability map of ommatidia location generated by the machine-learning module; (**C**) the anterior to posterior columns of detected ommatidia, separated by color and progressive dimming; (**D**) a contour map of ommatidia altitude (in micron, calculated relative to the lowest focal plane); (**E**) a contour map of estimated spacing between individual ommatidia (in micron), (**F**) ommatidia count in each column from posterior to anterior.

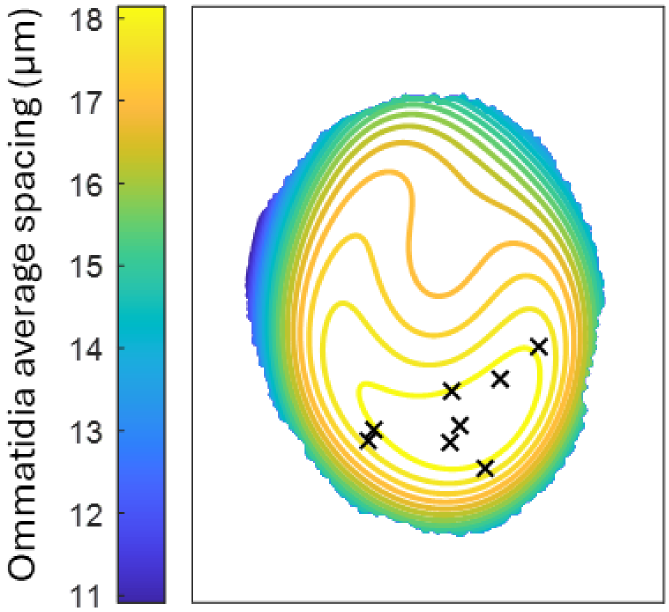

Fig. S7. Contour map of the average ommatidia spacing (in *μm*) in WTJ2 eyes. The map is projected onto the eye shown in Fig. S6. Black crosses indicate the locations of greatest inter-ommatidial distance (largest ommatidial diameter) for individual WTJ2 eyes; data from n=8 eyes (data from *Ramaekers et al., 2019)*.

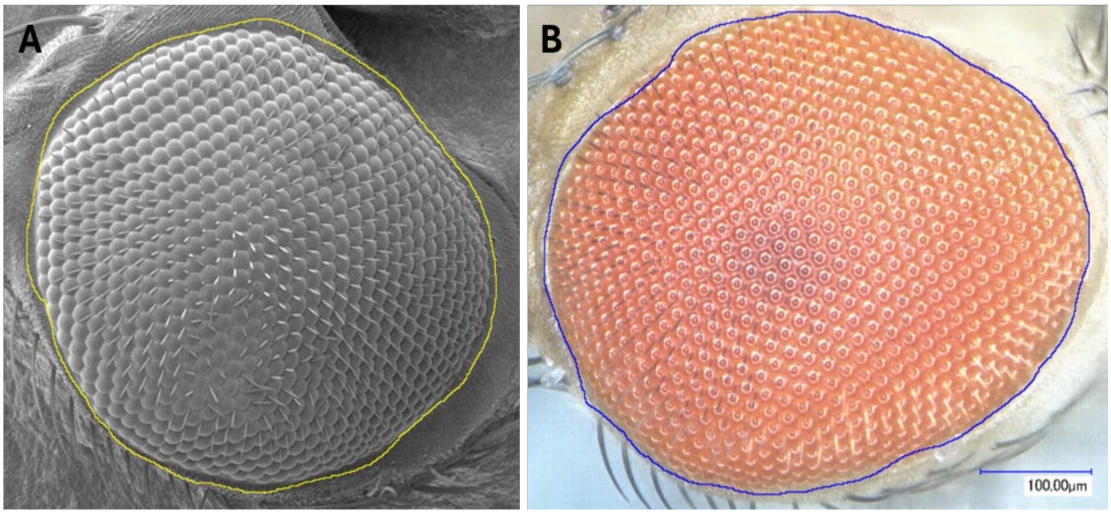

Fig. S8. Custom contours of Hikone-AS eyes. (A) Example of a SEM image with the hand-drawn yellow contour. (B) Example of a brightfield image with the hand-drawn blue contour.

Biology Open • Supplementary information

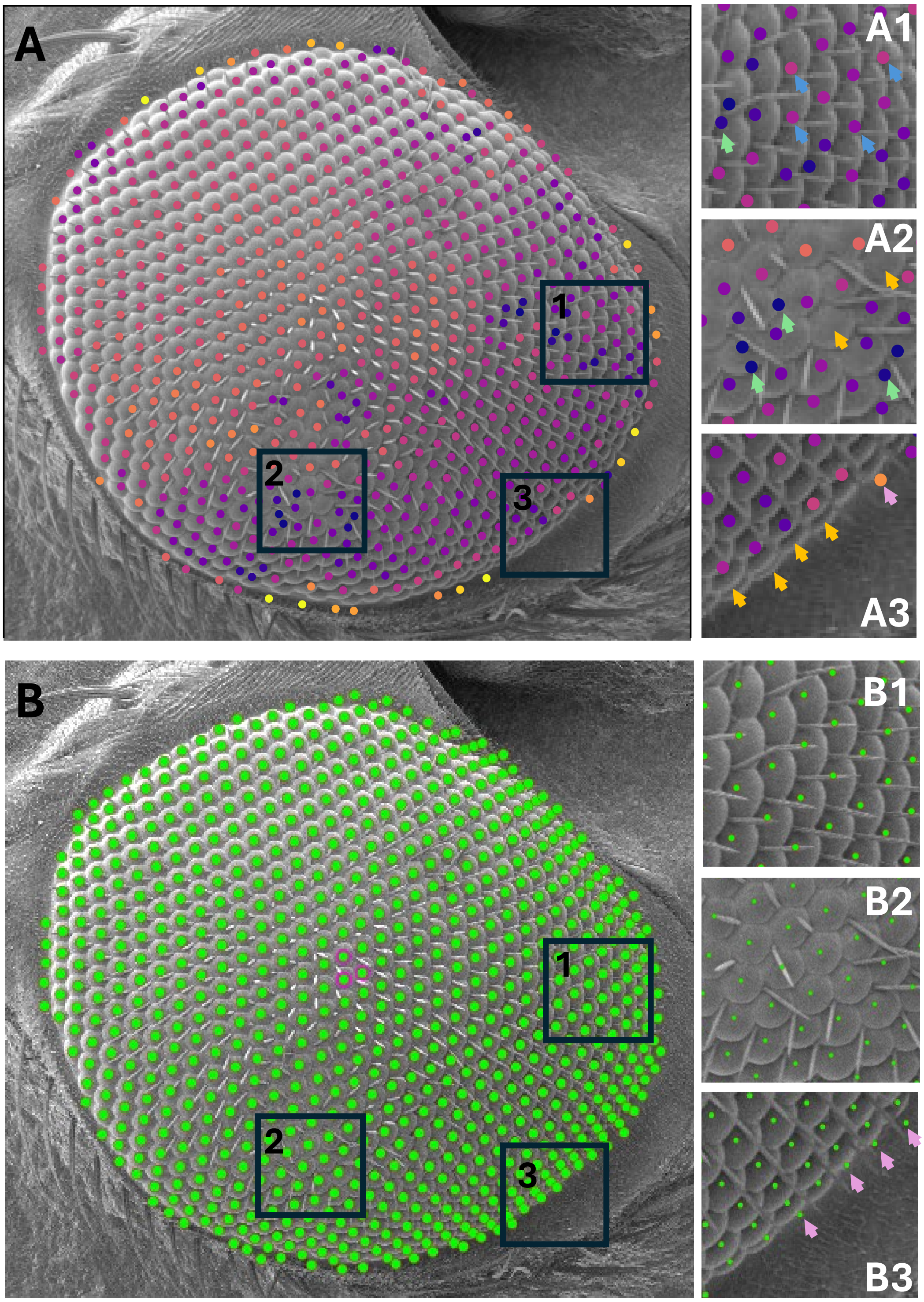

Fig. S9. Comparison of automatic segmentation function from Ommatidia Detection Algorithm (ODA) and EyeHex on a 2D SEM image. (A) Detected ommatidia (colored circles) with ODA projected onto the original images. Screenshot from ODA user interface. (B) Detected ommatidia (green circles) with EyeHex projected onto the original images. Screenshot from EyeHex user interface. Subpanels A1, A2, A3 and B1, B2, B3 are zoomed-in frames in the same regions of panel A and B respectively. The colored arrows in subpanels highlight different segmentation error types: underestimated ommatidia density (blue), false positives (green), false negatives (orange), over-segmentation outside the eye boundary (purple).

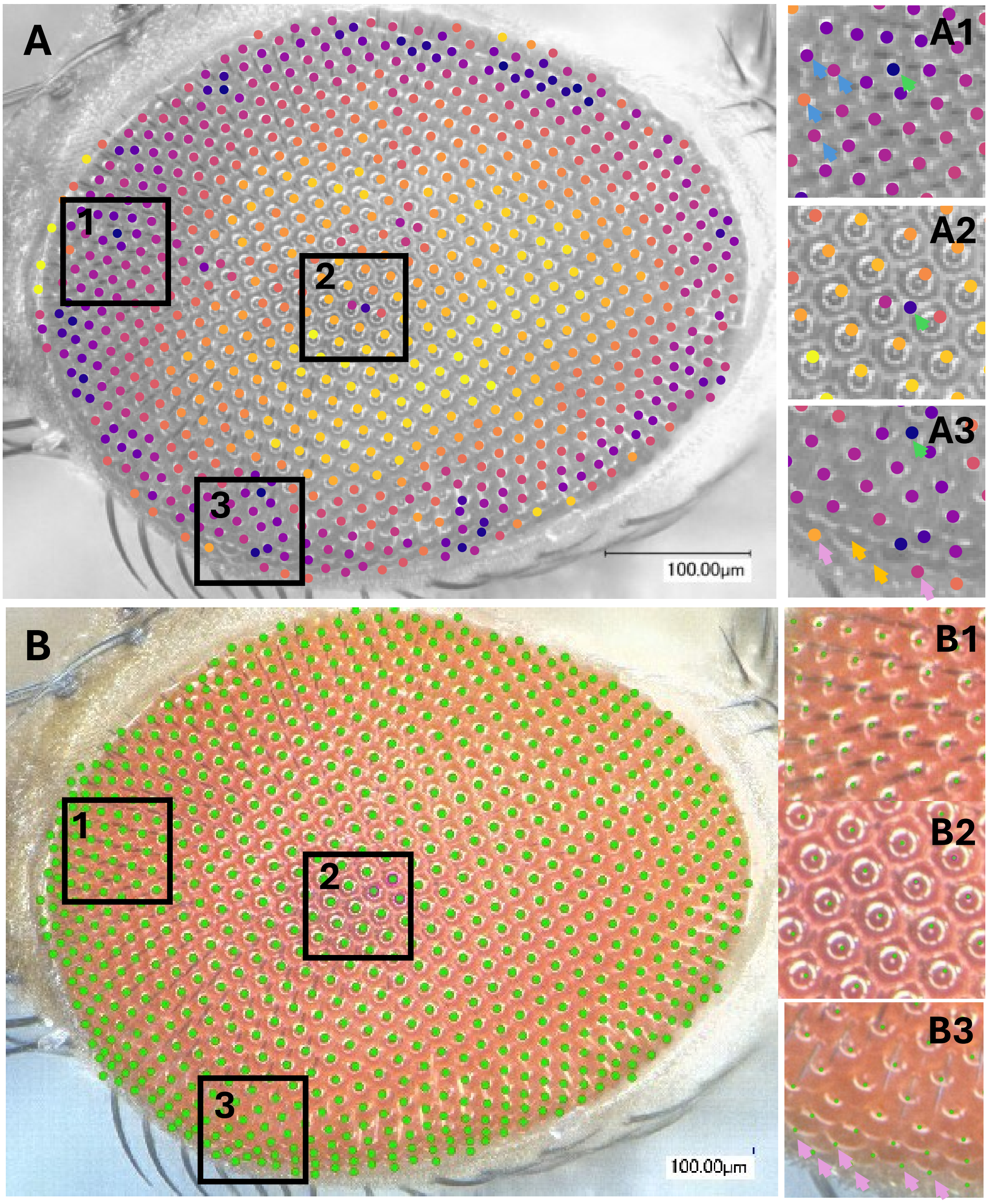

Fig. S10. Comparison of automatic segmentation function from Ommatidia Detection Algorithm (ODA) and EyeHex on a 2D brightfield image. (A) Detected ommatidia (colored circles) with ODA projected onto the original images. Screenshot from ODA user interface. (B) Detected ommatidia (green circles) with EyeHex projected onto the original images. Screenshot from EyeHex user interface. Subpanels A1, A2, A3 and B1, B2, B3 are zoomed-in frames in the same regions of panel A and B respectively. The colored arrows in subpanels highlight different segmentation error types: underestimated ommatidia density (blue), false positives (green), false negatives (orange), over-segmentation outside the eye boundary (purple).

Table S1. Ommatidia counts with EyeHex and ODA from SEM and brightfield images

Image type	n	EyeHex Pre-correction	ODA Pre-correction	EyeHex Post-correction*	Manual counts
SEM	6	819.3 ± 33.4	720.5 ± 39.7	741.2 ± 21.8	739.7 ± 21.8
Brightfield	12	831.1 ± 19.0	722.2 ± 17.4	752.7 ± 15.6	-

*Ommatidia counts (mean ± standard deviation) from SEM or brightfield images after automatic segmentation with EyeHex and ODA. Also shown are the final counts from EyeHex after manual correction and manual counts from SEM images (reused from Table 1 of main text). *The EyeHex final ommatidia counts are concluded with the eye regions defined by a trained classifier (using the full EyeHex protocol), rather by hand-drawn masks.*

Table S2.

Available for download at
https://journals.biologists.com/bio/article-lookup/doi/10.1242/bio.061962#supplementary-data

Table S3.

Available for download at
https://journals.biologists.com/bio/article-lookup/doi/10.1242/bio.061962#supplementary-data

Biology Open • Supplementary information
